# 

**Published:** 1889-10

**Authors:** 


					﻿$2.00 A YEAR IN ADVANCE.
Whole Number,
CCCXXXIX.
New Series.
©ctober, 1889.
VOL. XXIX.
No. 3.
Buffalo
Medical #/ Surgical
Journal.
Established 1845 by AUSTIN FLINT, NT. D.
^Editors:
THOS. LOTHROP, M. D.
W. W. POTTER, M. D.
Associate SE&itors:
FRANK HAMILTON POTTER, M. D.
WILLIAM C. KRAUSS, M. D.
JAMES WRIGHT PUTNAM, M. D.
JOHN PARMENTER, M. D.
BUFFALO:
1889.
Entered at the Post-Office at Buffalo, N. Y., as Second-class Mail Matter.
Times Printing House, Buffalo, N. V.
				

